# *Mitf* Involved in Innate Immunity by Activating Tyrosinase-Mediated Melanin Synthesis in *Pteria penguin*

**DOI:** 10.3389/fimmu.2021.626493

**Published:** 2021-05-20

**Authors:** Feifei Yu, Yishan Lu, Zhiming Zhong, Bingliang Qu, Meifang Wang, Xiangyong Yu, Jiayu Chen

**Affiliations:** ^1^ Fishery College, Guangdong Ocean University, Zhanjiang, China; ^2^ Faculty of Chemistry and Environmental Science, Guangdong Ocean University, Zhanjiang, China; ^3^ Ocean College, South China Agriculture University, Guangzhou, China

**Keywords:** *Mitf*, melanin, tyrosinase, innate immunity, *Pteria penguin*

## Abstract

The microphthalmia-associated transcription factor (MITF) is an important transcription factor that plays a key role in melanogenesis, cell proliferation, survival and immune defense in vertebrate. However, its function and function mechanism in bivalve are still rarely known. In this research, first, a *Mitf* gene was characterized from *Pteria penguin* (*P. penguin*). The *PpMitf* contained an open reading frame of 1,350 bp, encoding a peptide of 449 deduced amino acids with a highly conserved basic helix-loop-helix-leucine zipper (bHLH-LZ) domain. The PpMITF shared 55.7% identity with amino acid sequence of *Crassostrea gigas* (*C. gigas*). Tissue distribution analysis revealed that *PpMitf* was highly expressed in mantle and hemocytes, which were important tissues for color formation and innate immunity. Second, the functions of *PpMitf* in melanin synthesis and innate immunity were identified. The *PpMitf* silencing significantly decreased the tyrosinase activity and melanin content, indicating *PpMitf* involved in melanin synthesis of *P. penguin.* Meanwhile, the *PpMitf* silencing clearly down-regulated the expression of *PpBcl2* (B cell lymphoma/leukemia-2 gene) and antibacterial activity of hemolymph supernatant, indicating that *PpMitf* involved in innate immunity of *P. penguin.* Third, the function mechanism of *PpMitf* in immunity was analyzed. The promoter sequence analysis of tyrosinase (*Tyr*) revealed two highly conserved E-box elements, which were specifically recognized by HLH-LZ of MITF. The luciferase activities analysis showed that *Mitf* could activate the E-box in *Tyr* promoter through highly conserved bHLH-LZ domain, and demonstrated that *PpMitf* involved in melanin synthesis and innate immunity by regulating tyrosinase expression. Finally, melanin from *P. penguin*, the final production of *Mitf*-*Tyr*-melanin pathway, was confirmed to have direct antibacterial activity. The results collectively demonstrated that *PpMitf* played a key role in innate immunity through activating tyrosinase-mediated melanin synthesis in *P. penguin.*

## Introduction

Invertebrates lack highly evolved adaptive immunity system, and completely rely on innate immunity mediated by both cellular and humoral components to protect the host from microbial challenge (1, 2). Most invertebrates have several innate immune responses, of which melanization is an important humoral immune response (3, 4). By melanization, the melanin is largely synthesized and deposited in infected site for wound healing, phagocytosis, parasite entrapment and microbe killing (1, 5). Although there is no typical melanization observed in bivalve, the melanin and the enzymes involved in melanin synthesis are speculated to be important for innate immunity of bivalve (1, 6).

The microphthalmia-associated transcription factor (MITF) is a member of microphthalmia-associated transcriptional factor (MiT) family of transcription factors (7, 8). MITF acts as a central transcription factor to regulate the expression of tyrosinase (*Tyr*), an initial and rate-limiting enzyme of melanin synthesis, and controls the melanin production (9, 10). MITF also participates in immune defense by regulating lots of target genes in innate immune signaling pathway, such as tyrosinase, pthnoloxidase (*PO*), cathepsin K (CTSK) and B cell lymphoma/leukemia-2 (BCL2) (7, 11, 12). Although *Mitf* genes have been widely reported in vertebrates, the reports in bivalve are quite meager, only two *Mitf* genes have been identified from *Patinopecten yessoensis* and *Meretrix petechialis* so far (13, 14). It is not known whether *Mitf* performs a similar function in melanin synthesis and innate immunity of bivalve.

The winged pearl oyster *Pteria penguin* (*P. penguin*) is an important commercial bivalve cultivated in South Sea, and is used to produce high-quality sea pearls (15). *P. penguin* has pure black shell, suggesting the existence of abundant melanin. Our previous researches have confirmed that melanin determines the color formation of nacre of *P. penguin* (16, 17). It is worth studying whether melanin involves in innate immunity, and how melanin-synthesis related genes regulate the innate immune response of bivalve.

In this research, we characterized the new *Mitf* gene from *P. penguin*, and confirmed it played a crucial role in both melanin synthesis and innate immunity. Moreover, mechanism studies showed that *PpMitf* was involved in innate immunity by activating tyrosinase and motivating the biosynthesis of melanin. *Mitf*–*Tyr*–melanin pathway is an essential pathway in innate immunity of *P. penguin*

## Materials and Methods

### Experimental Animals and Arbutin Treatment

The *P. penguin* used in this research were cultivated in Weizhou Island in Beihai, Guangxi Province, China. Their shell length is 14 ± 1 cm, weighing 400 ± 50 g. They were held in circulating seawater at 25 ± 0.5°C for 5 days in lab prior to experiments. If necessary, the experimental individuals were immersed in 10 mM arbutin diluted with seawater for 7 days to inhibit tyrosinase activity.

### RNA Interference Experiment and Samples Collection

RNA interference was performed to identify the function of *PpMitf* gene. The *PpMitf*-siRNA1 was synthesized to silence the N-terminal conserved region, and *PpMitf*-siRNA2 was synthesized to silence the highly conserved HLH-LZ domain. The GFP-siRNA was synthesized from pEGFP-N3 plasmid as a negative control (NC) (primers as **Table 1**). In blank group, the experimental animals were cultivated with the recirculating seawater without any treatment. Double-stranded RNA (dsRNA) was synthesized with T7 High Efficiency Transcription Kit (TransGen, China) and purified with EasyPure RNA Purification Kit (TransGen, China). 100 μl of 1 μg/μl dsRNA were gently injected into adductor muscle of experimental individuals, and were injected again at the 5th day with the same dose to enhance the silencing effect. At the 8th day, the mantle was collected for RNA extraction, tyrosinase activity assay and melanin analysis. The hemolymph was collected from adductor muscle and immediately centrifuged at 800*g*, 4°C for 10 min to separate the hemocytes and supernatant. The hemocytes were harvested for tissue distribution analysis, and the supernatant was filter-sterilized (0.22 μm) for antibacterial activity. Each of the experimental groups contained five individuals.

**Table 1:** Primers used in the study.

Primer	Sequence (5′–3′)	Application
*PpMitf*-outer-F	GACCCAGATAGTCCCCTGTCAGCAGG	3′RACE
*PpMitf*-inner-F	AGCTTGATAGAGCCTACCCTTAATCAG	nest-3′RACE
*PpMitf-*outer-R	TTGAGGTTGATGTTGAGGTTGAGACTG	5′RACE
*PpMitf*-inner-R	AGACCATGTGCTTTCATTACCAACTCC	nest-5′RACE
UPM (Universal Primer)	TAATACGACTCACTATAGGGCAAGCAGTGGTATC AACGCAGAGT	RACE universal primer
NUP (Nested Universal Primer)	AAGCAGTGGTATCAACGCAGAGT	Nest-RACE universal primer
*PpMitf*-test-F	ATGCAGGACTCTGGAATCGAATATG	cDNA test
*PpMitf*-test-R	TCACAGCAAATCGTTCGATTCGGA	cDNA test
*PpMitf*-siRNA1-F	GCGTAATACGACTCACTATAGGGGACCATCAAAACCGAGACACAAGCA	RNAi
*PpMitf*-siRNA1-R	GCGTAATACGACTCACTATAGGGAAATTAGCTGGACAGGAAGAGGAGC	RNAi
*PpMitf*-siRNA2-F	GCGTAATACGACTCACTATAGGGGGACAGACAGAAGAAGGATAATCAC	RNAi
*PpMitf*-siRNA2-R	GCGTAATACGACTCACTATAGGGAGTTCATCTCGCTTTGAGGTTGATG	RNAi
*PpMitf*-pcDNA3.1-F	GTAGCTAGCATGCAGGACTCTGGAATCGA	Luciferase activity analysis
*PpMitf*-pcDNA3.1-R	GCTCTAGATCACAGCAAATCGTTCGATT	Luciferase activity analysis
GFP-siRNA-F	GATCACTAATACGACTCACTATAGGGATGGTGAGCAAGGGCGAGGA	RNAi
GFP-siRNA-R	GATCACTAATACGACTCACTATAGGGTTACTTGTAC AGCTCGTCCA	RNAi
*Tyr*-SP1	CAGTATAGTTAAGTCTGTACTGC	Genomic Walking
*Tyr*-SP2	GTAGATATTTGCAGGTATGAAAG	Genomic Walking
*Tyr*-SP3	GATCTGTGAGAGATATAAACTTC	Genomic Walking
*Tyr*-pro-F	GAAGAGCTCAAGACAGAATG	Promoter verification
*Tyr*-pro-R	CAGTATAGTTAAGTCTGTACTGC	Promoter verification
*Tyr*-pro-luc-F	CTTGCTAGCACTAATGGGACTCTAGCAGG	Luciferase activity analysis
*Tyr*-pro-luc-R	CGCAAGCTTAATCAAATTCCTAAAGCACT	Luciferase activity analysis
*PpTyr*-qPCR-F	CTCAGGGAAGGGATCAGCTT	qRT-PCR
*PpTyr*-qPCR-R	AGACCCTCTGCCATTACCAA	qRT-PCR
*PpMitf*-qPCR-F	TGTTACCTAAATCTGTTGATCCAG	qRT-PCR
*PpMitf*-qPCR-R	AAATTAGCTGGACAGGAAGAGGAG	qRT-PCR
*PpBcl2*-qPCR-F	TGAGGCACAGTTCCAGGATT	qRT-PCR
*PpBcl2*-qPCR-R	ACTCTCCACACACCGTACAG	qRT-PCR
*PpCdk2*-qPCR-F	TGGATTTGCTCGGACACTTG	qRT-PCR
*PpCdk2*-qPCR-R	TCTACTGCCCTGCCATACTT	qRT-PCR
β-actin-F	CGGTACCACCATGTTCTCAG	qRT-PCR
β-actin-R	GACCGGATTCATCGTATTCC	qRT-PCR

### RNA Isolation and cDNA Synthesis

Total RNA were isolated from about 2 g of mantle, gill, adductor muscle, digestive diverticulum, foot, gonad and hemocytes of *P. penguin* using RNeasyMini Kit (Qiagen, USA). The single strand cDNA was synthesized from total RNA using a Superscript II polymerase kit (TransGen, China) and used as templates of Real-Time PCR. The random primers was employed for cDNA synthesis.

### The cDNA Cloning and Sequence Analysis

The full-length cDNA of *Mitf* was obtained with SMART RACE cDNA Amplification Kit (Clontech, USA) and Advantage 2 cDNA Polymerase Mix (Clontech, USA). The specific primers (*PpMitf*-outer-F and *PpMitf*-outer-R) were designed based on the partial sequence from the transcriptome, and were used to amplify the 3’ and 5’ sequences. The nested-PCR was performed to enrich the specific DNA band using *PpMitf*-inner-F and *PpMitf*-inner-R. The nested-PCR program was conducted as follows: 94°C for 4 min, 35 cycles (94°C for 30 s, 57°C for 30 s and 72°C for 1 min 20 s in each cycle) and 72°C for 10 min. The test-PCR was employed to certify the nucleotide sequence using *PpMitf*-test-F and *PpMitf*-test-R. All primers were showed in **Table 1**.

The *Mitf* cDNA was analyzed using the BLAST program, and the open reading fragment (ORF) was identified using ORF Finder. The signal peptide was predicted by SignalP. Multiple sequences were aligned using Clustal W, and phylogenetic tree was constructed using MEGA 6. The protein molecular weight and theoretical pI were analyzed by programs online (http://web.expasy.org/cgibin/protparam/protparam).

### Quantitative Real-Time PCR (qRT-PCR) Analysis

The Real-Time PCR was performed by the Applied Biosystems 7500/7500 Fast Real-time System (ABI, USA) following the manufacturer’s protocol of DyNAmo Flash SYBR Green qPCR Kit (Thermo scientific, USA). The reaction was run in a 10 μl volume containing 20 ng of cDNA, 0.3 μM of each primer and 5 μl SYBR green Master Mix. The PCR parameters were 95°C for 2 min, followed by 38 cycles of 95°C for 5 s, 58°C for 20 s and 72°C for 20 s. The specific primers were listed in **Table 1**, and β-actin was used as internal control. The 2^−ΔΔCT^ method was applied to calculate the relative expression levels of genes. Each reaction was repeated in triplicate.

### Tyrosinase Activity Assay

Tyrosinase activity assays were performed following the previous reports with minor modification (17–19). 1 g mantle tissue was homogenized in 3 ml of 0.1 mol/L Phosphate Buffered Saline (PBS, pH 6.8), and was centrifuged at 12,000*g* for 10 min to obtain the supernatant (about 1 ml). Then, 0.5 ml of 5 mmol/L 3, 4-dihydroxyphenylalanine (L-DOPA) was mixed with all the supernatant, and incubated at 37°C for 30 min. The absorbance of the mixture was recorded at 475 nm. The tryosinase activity was defined as increased or decreased absorbance in 30 min at 475 nm.

### Isolation and Oxidation of Total Melanin

The melanin was isolated from mantle of *P. penguin* and oxidized as follows (15). 1 g mantle sample was finely homogenized on ice, mixed with 15 ml PBS (pH 7.4) with 2% (m/V) papain (J&K, China), and incubated at 55°C for 20 h. The precipitate was obtained by centrifuging at 12,000*g* for 10 min, and then was successively washed with 2 ml mineral ether, ethanol and water. After that, the obtained black precipitate was raw melanin production.

8.6 ml of 1 mol/L K_2_CO_3_ and 0.8 ml of 30% H_2_O_2_ were used to dissolve and oxidize the raw melanin. The mixture was heated at 100°C for 20 min and cooled down to room temperature. The residual H_2_O_2_ was decomposed by 0.4 ml of 10% Na_2_SO_3_, and 6 mol/L HCl was then added to adjust pH to 1.0. The mixture was centrifuged at 8,000*g* for 10 min to get the supernatant, which then was extracted using 70 ml of ether and dried to crystalline residue. Finally, crystalline residues were redissolved in mobile phase or water, and were filtered through 0.45 μm nylon membrane (Millipore, USA) before using.

### LC-MS/MS Assay of Melanin

The liquid chromatograph-tandem mass spectrometer (LC-MS/MS) was employed to detect the content and component of melanin (16, 20). The chromatographic separation was performed using an Acquity ultraperformance liquid chromatography (UPLC) system (Waters, USA) with a Waters ACQUITY UPLC HSS T3 (2.1 × 50 mm, 1.7 μm particle size). The mobile phase A was 0.1% of formic acid/deionized water (*v*/*v*), and mobile phase B was 0.1% of formic acid/methanol (*v*/*v*). The ratio of mobile phases A and B was 9:1 in the first 3 min, and 1:9 in the last 3 min. It kept 6 min in one cycle. Analyses were performed at 40°C at a flow rate of 0.3 ml/min. As the MS/MS detection, a Xevo TQ triple quadrupole mass spectrometer was operated in positive electrospray ionization (ESI) mode. The Mass spectrometer parameters were as follows: The source temperature was 150°C, and desolvation temperature was 550°C. The cone gas flow, desolvation gas flow and collision gas flow were 50 L/h, 1.100 L/h and 0.14 ml/min (argon), respectively. The analytes were monitored in multireaction monitoring mode (MRM).

### Genome Walking

The promoter region of *PpTyr* was cloned using the Universal Genome Walker 2.0 Kit (Clontech, USA). The Genome Walker libraries were constructed using the genomic DNA, which was extracted from *P. penguin* by E.Z.N.A. Tissue DNA Kit (Omega, America). Three primers (*Tyr*-SP1, *Tyr*-SP2 and *Tyr*-SP3) were designed to amplify the single DNA fragments of *Tyr*. The PCR program was conducted as follow: 94°C for 1 min, 98°C for 1 min, five cycles (94°C for 30 s, 62°C for 1 min and 72°C for 3 min in each cycle), 15 cycles (94°C for 30 s, 25°C for 3 min, 72°C for 3 min; 94°C for 30 s, 62°C for 1 min, 72°C for 3 min; 94°C for 30 s, 44°C for 1 min, 72°C for 3 min) and 72°C for 10 min. Then the *Tyr*-pro-F and *Tyr*-pro-R were used to verify the amplified sequence (**Table 1**).

### Plasmids Construction

The Mitf-pcDNA3.1 plasmid was made by inserting the *Mitf* ORF sequence into pcDNA 3.1 vector with NheI and XbaI. The primers *PpMitf*-pcDNA3.1-F and *PpMitf*-pcDNA3.1-R were used to amplify the *Mitf* ORF (**Table 1**). The Mitf-△HLHLZ sequence, which was HLHLZ-deleted*-Mitf* ORF sequence (deletion from 854 to 1,022), was synthesized and inserted into the pcDNA3.1 vector with NheI and XbaI to construct the Mitf-△HLHLZ-pcDNA3.1 plasmid.

The *Tyr* promoter-driven luciferase reporter construct (Tyr-promoter-Luc) was made by inserting the whole *Tyr* promoter region (from −1,943 to −1) in front of the luciferase reporter gene in pGL3-Basic vector. The primers *Tyr*-pro-luc-F and *Tyr*-pro-luc-R were used to amplify the *Tyr* promoter fragment (**Table 1**). The Ebox-deleted-promoters were synthesized and inserted into the pGL3-Basic vector to construct the Tyr-△Ebox1-promoter-Luc (deletion from −1,767 to −1,761), Tyr-△Ebox2-promoter-Luc (deletion from −1,613 to −1,607) and Tyr-△Eboxes-promoter-Luc plasmids (deletion from −1,767 to −1,607). The NheI and HindIII were employed to digest the DNA fragment and pGL3-Basic vector.

### Luciferase Activity Assay

To analyze the *Tyr* promoter activity, 293T cells were grown in DMEM medium supplemented with 10% fetal calf serum (FCS) at 37°C in incubator with CO_2_. 0.4 μg of Tyr-promoter-Luc vector and 0.04 μg pRL-cmv vector were diluted in 50 μl DMEM and mixed with 1 μl of Lipofectamine 2000 (Invitrogen, USA) in 50 μl DMEM. After incubation for 5min at room temperature, the 100 μl of mixture was transfected into cells in 24-well plate. 0.4 μg of pGL3-Basic vector and 0.04 μg pRL-cmv vector were transfected as control. After 48h, the cells were collected and lysed using the Dual-Luciferase Reporter Assay System (Promega, America). The fluorescence intensity was measured by Junior LB9509 Luminometer. Luciferase activities were presented by relative light units (RLU) of firefly fluorescence to Renilla fluorescence. Each independent experiment was repeated five times.

To analyze the regulation of *Mitf* on *Tyr* promoter, the transfected 293T cells were cotransfected with 0.4 μg *Mitf*-pcDNA3.1, 0.4 μg of Tyr-promoter-Luc vector and 0.04 μg pRL-cmv vector, 0.4 μg pcDNA3.1 plasmid was used as control. To confirm the function of conserved HLH-LZ domain in MITF, Mitf-△HLHLZ-pcDNA3.1, Tyr-promoter-Luc and pRL-cmv plasmids were cotransfected into 293T cells. To elaborate the role of E-box in *Tyr* promoter, the *Mitf*-pcDNA3.1, Tyr-△Ebox-promoter-Luc plasmid and pRL-cmv vector were cotransfected.

### Western Blot

The equal amounts of transfected 293T cells were collected and used for proteins extraction with TRIZOL reagent (Invitrogen, USA) according to the previous report (21). The protein extracts were separated on the 12% SDS-PAGE gel and electrophoretically transferred to a PVDF membrane (Millipore, USA). The membrane was blocked with 3% BSA (Bovine serum albumin)/PBS (phosphate buffer saline) for whole night, and then was washed for three times by PBST, each for 10 min. The membrane was incubated with primary antibody in 1% BSA/PBS for 1.5 h, washed three times and then incubated with secondary antibody for 1 h at room temperature. After another three 10-min washes with PBST, the membrane was stained with NBT/BCIP staining system (Sigma-Aldrich, USA) and by detected in dark. The anti-Flag antibody (Yeasen, China) was used as primary antibody with dilution ratio of 1:1,000. The anti-actin antibody (Yeasen, China) was used as an internal control with dilution ratio of 1:4,000. The HRP-conjugated goat anti-rabbit IgG (Sigma-Aldrich, USA) was used as secondary antibody at 1:4,000.

### Antibacterial Activity Assay of Hemolymph Supernatant

The antibacterial activity of the hemolymph supernatant was assayed using the method described previously (22, 23). The protein concentrations in haemolymph supernate from NC, siRNA1 and siRNA2 groups were adjusted to 1.0 mg/ml using Nanodrop spectrophotometers (Thermo scientific, USA). The 50 μl of sterile hemolymph supernatant was mixed with 50 μl of *E. coli* containing pMD-18T vector (TaKaRa, Japan) at a density of 1 × 10^6^ colony forming units (CFU)/ml, and incubated at 37°C for 30 min with shaking. Then, 50 μl of mixture was diluted with 250 μl LB medium, and pipetted into a sterile 96-well plate. The plate was incubated at 37°C for 12 h, and the absorbance at 600 nm was measured at intervals of 30min. The time when OD600 absorbance of NC group reached the maximum was recorded, and half of the time was defined as T50. The OD600 value at T50 was used to represent the anti-bacterial activity of hemolymph supernatant. Five individuals were used in each treatment group.

### Antibacterial Activity Assay of Melanin Oxidation Products

The *E. coli* with pMD-18T vector (TaKaRa, Japan) was cultured to a density of 1 × 10^6^ CFU/ml (24). The melanin of 1 g mantle from NC, siRNA1 and siRNA2 groups was extracted, oxidized, filtered and resolved in 50 μl sterilized water. Then, 50 μl melanin oxidation production was mixed with 150 μl *E. coli* with ampicillin resistance, and shaken for 0.5 h at 37°C. In melanin-addition groups, 0.1 g melanin (J&K, China) was oxidized, filtered, resolved and added into the mixture. Then, 100 μl mixture was evenly spread on plates with LB medium and 50 μg/ml ampicillin. After incubation at 37°C for 24 h, the number of visible colonies was counted.

### Statistical Analysis

Analysis of Variance (ANOVA) was performed to determine the significant differences in different samples (*n* = six replicates) by SPSS (Version 17.0, Chicago, IL, USA). Data were shown as mean± SD. *(*P <*0.05) meant significant difference, and **(*P <*0.01) meant highly significant difference.

## Results

### Cloning and Sequence Analysis of *Mitf* cDNA in *P. penguin*

**Figure 1:**
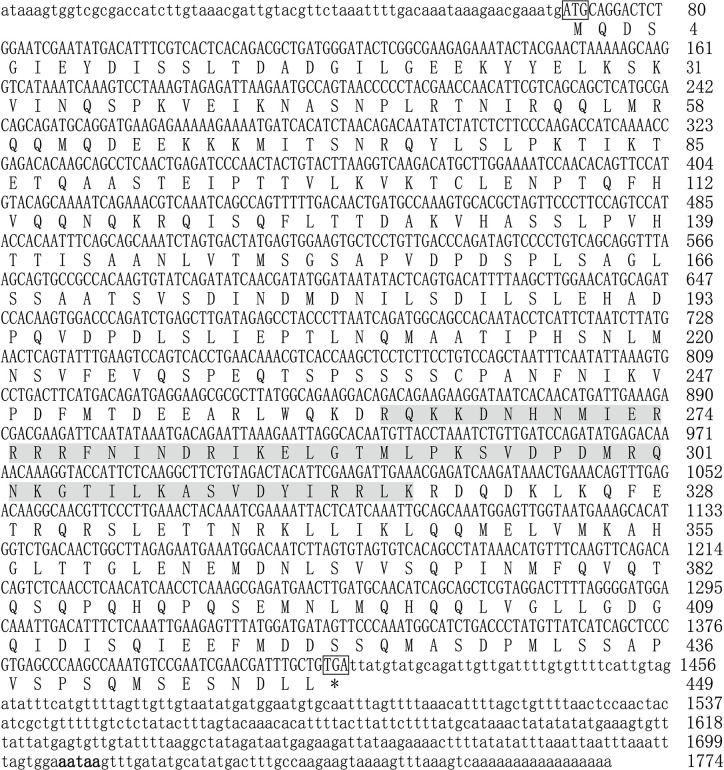
The full-length nucleotide and deduced amino acid sequences of *PpMitf*. The ORF sequence was displayed in uppercase, the 5’ UTR and 3’ UTR sequences were displayed in lowercase. The initiation codon (ATG) and termination codon (TGA) were boxed. The putative bHLH-LZ domains were boxed in gray. The signal peptide (aataa) was marked in boldface. * meant no amino acid was coded.

The complete coding sequence of *Mitf* in *P. penguin* was cloned from mantle by RACE-PCR and named as *PpMitf* (Genbank accession no. MN296415). The complete nucleotide sequence of *PpMitf* was 1,774 bp in length, containing a 1350-bp open reading frame (69–1,418), a 68-bp 5′-untranslated region (UTR) and a 356-bp 3’-UTR with a typical signal sequence (AATAA) located upstream of poly (A) tail (**Figure 1**). The ORF encoded 449 deduced amino acids without a signal peptide. The predicted polypeptide sequence contained a basic helix-loop-helix-leucine zipper (bHLH-LZ) domain, which recognized with E-box or M-box of downstream genes. The deduced molecular mass of *PpMitf* protein was 50.5 kDa with a theoretical isoelectric point (pI) of 5.34.

### Multiple Sequence Alignment and Phylogenetic Analyses

**Figure 2:**
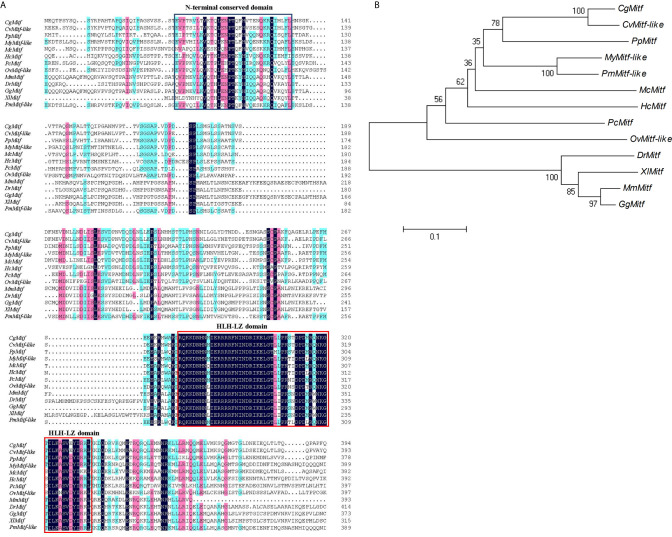
Sequence alignment and phylogenetic analysis. **(A)** Multiple sequence comparison of *Mitf* amino acid sequences in various species, including *Pteria penguin* (*PpMitf*, MN296415), *Crassostrea gigas* (*CgMitf*, XP_011447560.2), *Crassostrea virginica* (*CvMitf-like*, XP_022298291.1), *Mizuhopecten yessoensis* (*MyMitf-like*, XP_021364176.1), *Pecten maximus* (*PmMitf-like*, XP_033745263.1), *Mytilus coruscus* (*McMitf*, CAC5409749.1), *Hyriopsis cumingii* (*HcMitf*, QBG58751.1), *Pomacea canaliculata* (*PcMitf*, XP_025106304.1), *Octopus vulgaris* (*OvMitf-like*, XP_029634800.1), *Danio rerio* (*DrMitf*, NP_571922.2), *Xenopus laevis* (*XlMitf*, NP_001165646.1), *Gallus gallus* (*GgMitf*, BAA25648.1), *Mus musculus* (*MsMitf*, AAF81266.2). The black background showed highly conserved amino acids, the green and pink background showed similar amino acids. HLH-LZ domain was boxed in red, and N-terminal conserved domain was boxed in blue. **(B)** Phylogenetic tree of *Mitf* genes. The phylogenetic tree was constructed using neighbor-joining method. Numbers at the nodes represented the bootstrap values determined by bootstrap analysis of 1,000 replicates. The scale bar indicated the number of amino acid substitutions per site.

The DNAMAN6 software (Lynnon Biosoft, Canada) was used to determine the homology among *Mitf* gene from different species. The *PpMitf* shared the highest sequence similarity (55.7%) with *Mitf* gene of *Crassostrea gigas*, and 53.7, 51.6 and 51.3% sequence similarity with *Mitf*-*like* genes of *Crassostrea virginica*, *Pecten maximus* and *Mizuhopecten yessoensis*, respectively. The amino acid sequence comparison showed a highly conserved basic HLH-LZ domain among mollusks, fish, amphibians, birds and mammals. Another relatively conserved region was in the N-terminal, and named as N-terminal conserved domain. (**Figures 1** and **2A**)

To understand the evolutionary relationships among *PpMitf* and that of other species, the phylogenetic tree was constructed using MEGA7 (**Figure 2B**).The *PpMitf* was located in one clade with *Mitf* protein of *C. gigas* and *Mitf-like* protein of *C. virginica*, indicating that they were the most closely related homologs. Moreover, seven *Mitf* genes of bivalves, including *P. penguin*, *C. gigas*, *C. virginica*, *M. yessoensis*, *Hyriopsis cumingii*, *P. maximus* and *Mytilus coruscus*, were contained in a close cluster. The *Mitf* genes of *Pomacea canaliculata* and *Octopus vulgaris* showed high homology with bivalves. On the other hand, all *Mitf* genes of vertebrates referred, including *Danio rerio*, *Xenopus laevis*, *Gallus gallus* and *Mus musculus*, were grouped into a big clade, and showed low homology with *PpMitf* gene.

### PpMitf Expression Profile in Different Tissues

**Figure 3:**
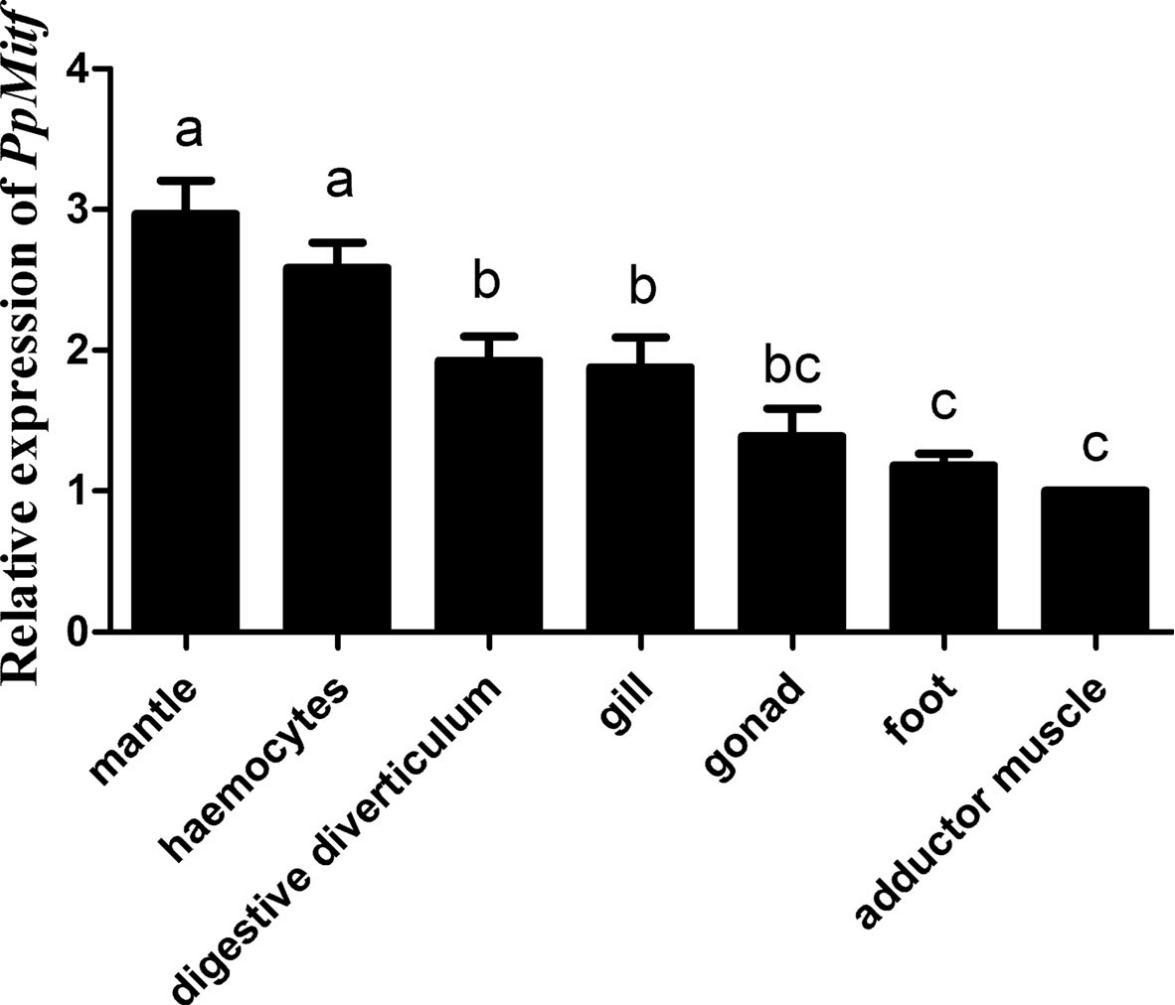
The expression of *PpMitf* in different tissues was validated by qRT-PCR. The abundance of *PpMitf* mRNA was normalized to that of β-actin of *P. penguin*. The data were represented as mean ± SD (*N* = 5). Different letters (a, b, bc and d) meant significant difference among these columns (*P <*0.05).

Using the qRT-PCR, the *PpMitf* mRNA levels from various tissues were investigated (**Figure 3**). *PpMitf* gene showed the highest expression levels in mantle and hemocytes, higher levels in gill and digestive diverticulum, and the lowest levels in adductor muscle, foot and gonad. Since *PpMitf* was mainly expressed in the mantle, which was responsible for melanin synthesis, nacre formation and innate immune response, the mantle was then used for gene expression, tyrosinase activity and melanin content analysis.

### PpMitf Silencing Inhibited Tyrosinase Activity

**Figure 4:**
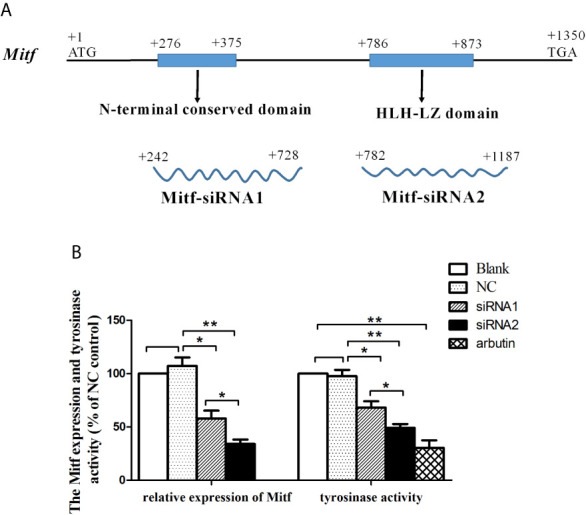
The effect of *Mitf* silencing on *Tyr* expression and tyrosinase activity. **(A)** The position and length of siRNA1 and siRNA2 **(B)** The *PpMitf* expression and tyrosinase activity analysis. The samples were from blank group, NC group (GFP-siRNA), *PpMitf*-siRNA1 and *PpMitf*-siRNA2 group. The β-actin of *P. penguin* was used as an internal control in qRT-PCR. The arbutin group was used as positive control in tyrosinase activity analysis. The data were represented as mean ± SD (*N* = 5). *P <0.05; **P <0.01; No signal means no difference.

RNA interference was conducted to examine the role of *Mitf* in melanin synthesis of *P. penguin.* The *PpMitf*-siRNA1 and *PpMitf*-siRNA2 were used to specifically silence the N-terminal conserved region and the HLH-LZ domain (**Figure 4A**). The *PpMitf* mRNA levels were measured by qRT-PCR after RNAi. **Figure 4B** showed that the *PpMitf* transcripts were down-regulated by 42.1% (*P <*0.05) and 65.9% (*P <*0.01) in siRNA1 and siRNA2 groups compared with the negative control (NC) group, indicating RNA interference produced a good silencing effect of *PpMitf* mRNA.

Tyrosinase activity is regarded as a marker of melanin biosynthesis because of its role as a key rate-limiting enzyme. The tyrosinase activity was represented by change in absorbance owing to the conversion of dopaquinone to dopachrome. The tyrosinase activity was significantly decreased by 30.2% through silencing N-terminal conserved domain (siRNA1 group) (*P <*0.05) and by 49.6% through silencing bHLH-LZ domain (siRNA2 group) (*P <*0.01) compared to NC group (**Figure 4B**). In positive control group, the experimental individuals were immersed in 10 mM arbutin, a typical tyrosinase activity inhibitor, and their tyrosinase activities were analyzed. The tyrosinase activity was significantly inhibited by 68.8% after arbutin treatment compared to blank group (*P <*0.01). This results indicated that the *PpMitf* silencing could inhibit tyrosinase activity in *P. penguin.*

### *PpMitf* Silencing Reduced Melanin Content

**Figure 5:**
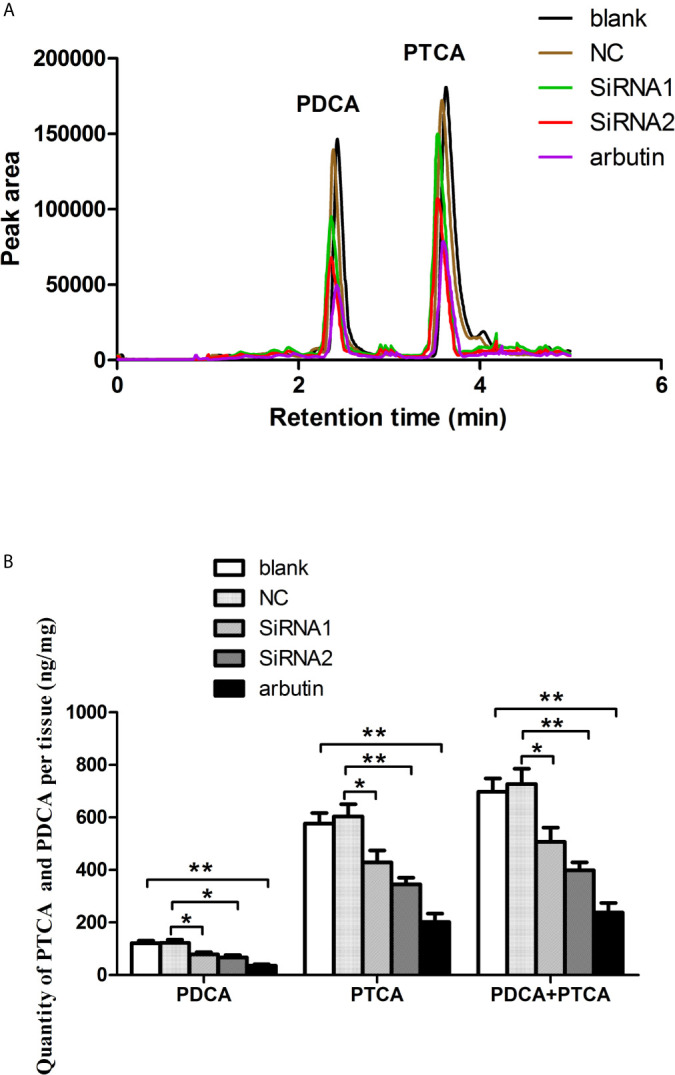
The content of melanin from samples in blank, NC, RNAi groups and arbutin group using LC-MS/MS analysis. **(A)** HPLC (High Performance Liquid Chromatography) chromatograms. **(B)** The content of PDCA and PTCA. The data were represented as the mean ± SD (*N* = 5). *P <0.05; **P <0.01.

After RNA interference, the qualitative and quantitative analysis of melanin were performed by LC-MS/MS. The mass spectrometry analysis verified that the main alkaline oxidation products of melanin from *P. Penguin* were pyrrole-2, 3-dicarboxylic acid (PDCA) and pyrrole-2, 3, 5-tricarboxylic acid (PTCA), with molecular weight at 156 and 199 g/mol. The quantitative analysis was measured based on the peak area of PDCA and PTCA, which appeared at 2.42 and 3.62 min (**Figure 5A**). The PDCA content was reduced by 35.9% through N-terminal conserved domain knockdown (siRNA1 group), and 48.5% through bHLH-LZ domain knockdown (siRNA2 group) (*P <*0.05). Similarly, the PTCA content was reduced by 29.1% by siRNA1 (*P <*0.05) and 42.8% by siRNA2 (*P <*0.01). The total content of PDCA and PTCA was clearly decreased by 30.2% through N-terminal conserved domain knockdown (*P <*0.05), by 45.2% through HLH-LZ domain knockdown (*P <*0.01), and by 65.9% through arbutin treatment (*P <*0.01) (**Figure 5B**). The data indicated that *PpMitf* regulated melanin synthesis in *P. penguin.*

### *Mitf* Silencing Inhibited the Transcription of Tyr, Cdk2 and Bcl2 in P. penguin

**Figure 6:**
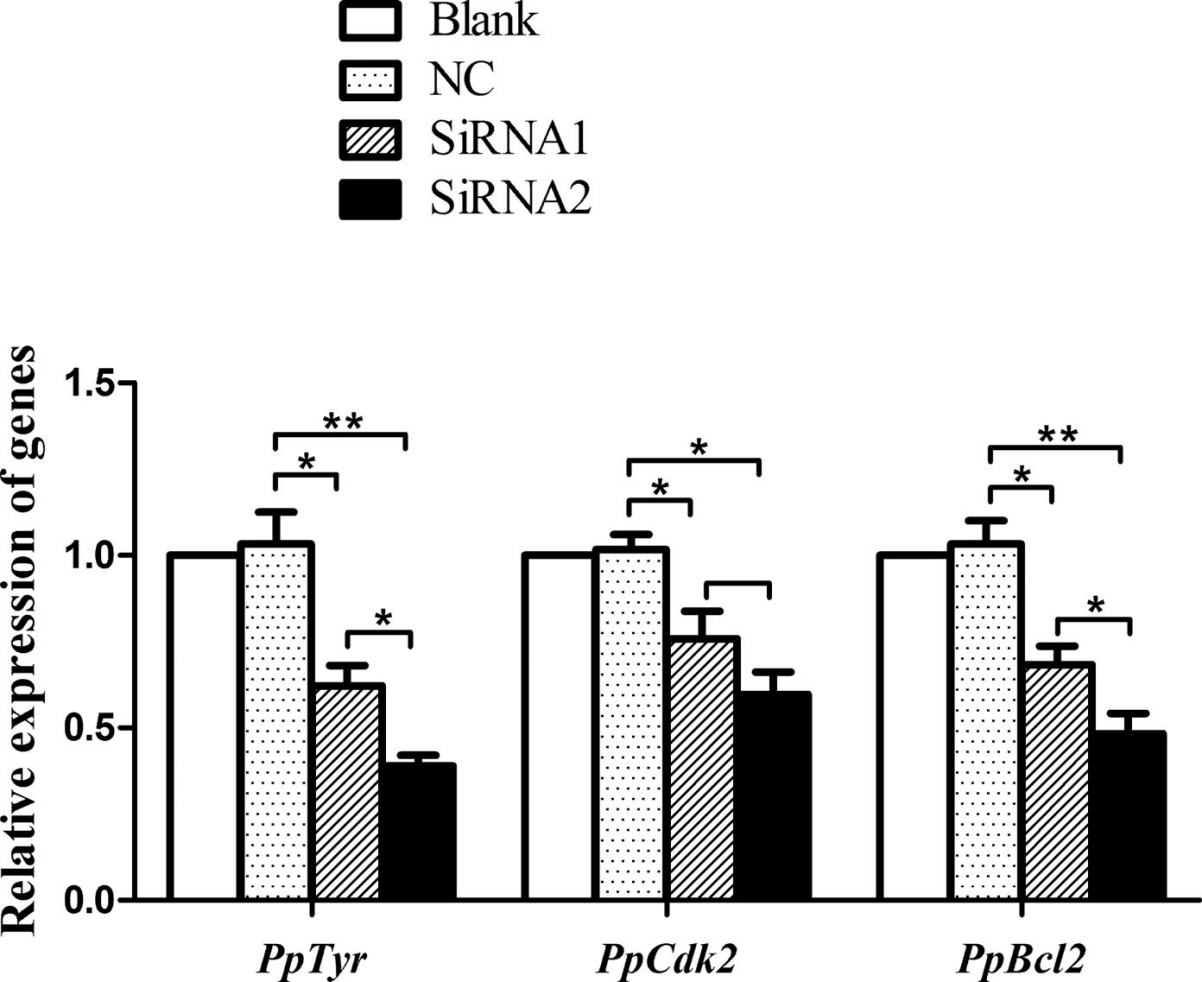
Transcriptional levels of *PpTyr*, *PpCdk2* and *PpBcl2* after *PpMitf* RNAi. The qRT-PCR was performed using samples from blank, NC, siRNA1 and siRNA2 group. The abundance of mRNA was normalized to that of β-actin of *P. penguin*. The data were represented as the mean ± SD (*N* = 5). *P <0.05; **P <0.01.

Since *PpMitf* silencing significantly reduced the tyrosinase activity and melanin content, we speculated that *PpMitf* silencing might inhibit the expression of tyrosinase gene in *P. penguin*. To prove this point, qRT-PCR was employed to detect the transcript level of *PpTyr.* As expected, the *PpTyr* mRNA was significantly down-regulated by 37.9% (*P <*0.05) by *Mitf*-siRNA1 and 61.0% (*P <*0.01) by *Mitf*-siRNA2 (**Figure 6**). This suggested that *PpMitf* took part in melanin synthesis by regulating the expression of *Tyr* in *P. penguin.*

In vertebrate, *PpMitf*, as a central transcriptional factor, controls the differentiation, growth and survival of melanocyte *via Tyr*, *Cdk2* (cyclin-dependent kinase 2) and *Bcl2*, respectively (25). So the *PpCdk2* and *PpBcl2* transcripts were analyzed after *PpMitf* silencing. The siRNA1 inhibited *PpCdk2* mRNA by 24.2% (*P <*0.05), and the siRNA2 inhibited *PpCdk2* by 40.3% (*P <*0.05). The *PpBcl2* mRNA was suppressed by 31.7% (*P <*0.05) and 51.6% (*P <*0.01) by siRNA1 and siRNA2 (**Figure 6**). These data suggested that *PpMitf* was capable of regulating the expression of *Cdk2* and *Bcl2* in *P. penguin*, similar to that of vertebrate.

### Sequence Characterization of *Tyr* promoter

**Figure 7:**
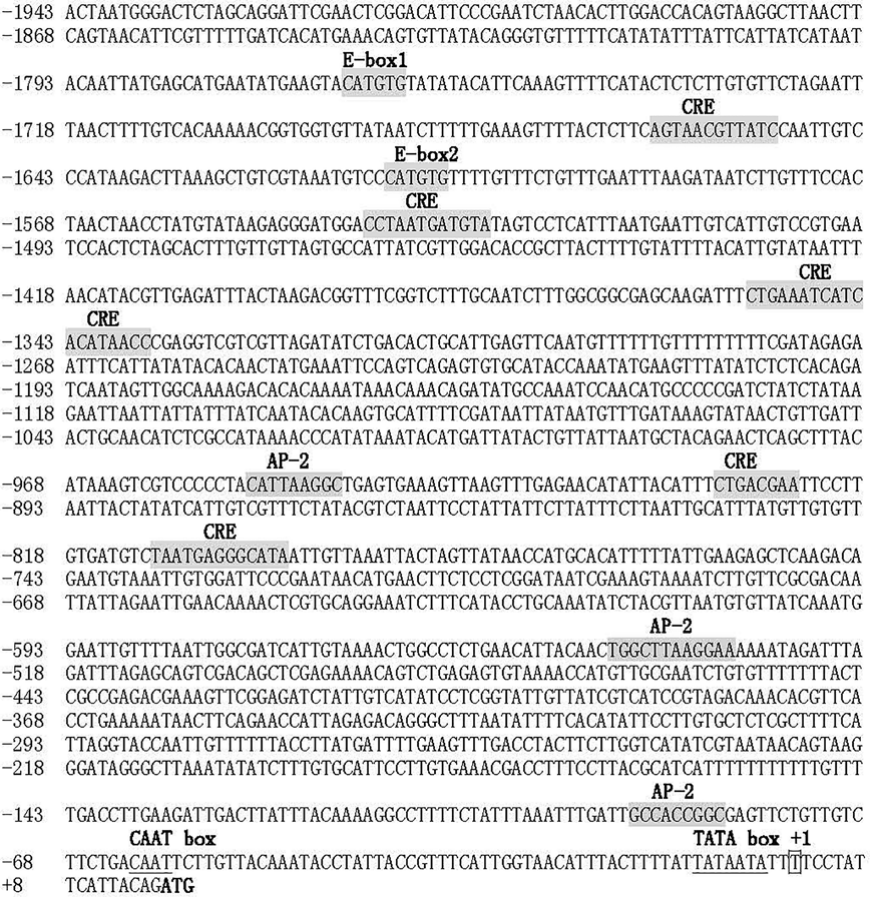
Sequence of the *Tyr* promoter. The putative transcriptional start site was indicated with box, and the initiation codon was bolded. The two conserved E-box, six CRE sites, two AP-2 sites were shaded in gray. The putative CAAT box and TATA box were underlined.

To analyze the expression regulation of *PpTyr*, the upstream promoter sequence of *PpTyr* was amplified by standard PCR and genome walking. As shown in **Figure 7**, a 1,959-bp genomic sequence upstream of initiation codon (ATG) was amplified by special primers. The transcriptional start site of *Tyr* gene, located 16 bp upstream from the ATG, was designated as position +1. The 1,943-bp sequence upstream of the transcriptional start site was considered as a putative promoter. Sequence analysis of the promoter revealed two typical E-box (CATGTG) elements, recognized by bHLH-LZ transcription factors, were located at positions from −1,767 to −1,761 and from −1,613 to −1,607. In addition, the tyrosinase promoter contained six putative cAMP response element (CRE) and three putative activating protein 2 (AP-2) binding sites, both of which were thought to response to intracellular Camp (26).

### *PpMitf* Activated the Expression of *PpTyr*

**Figure 8:**
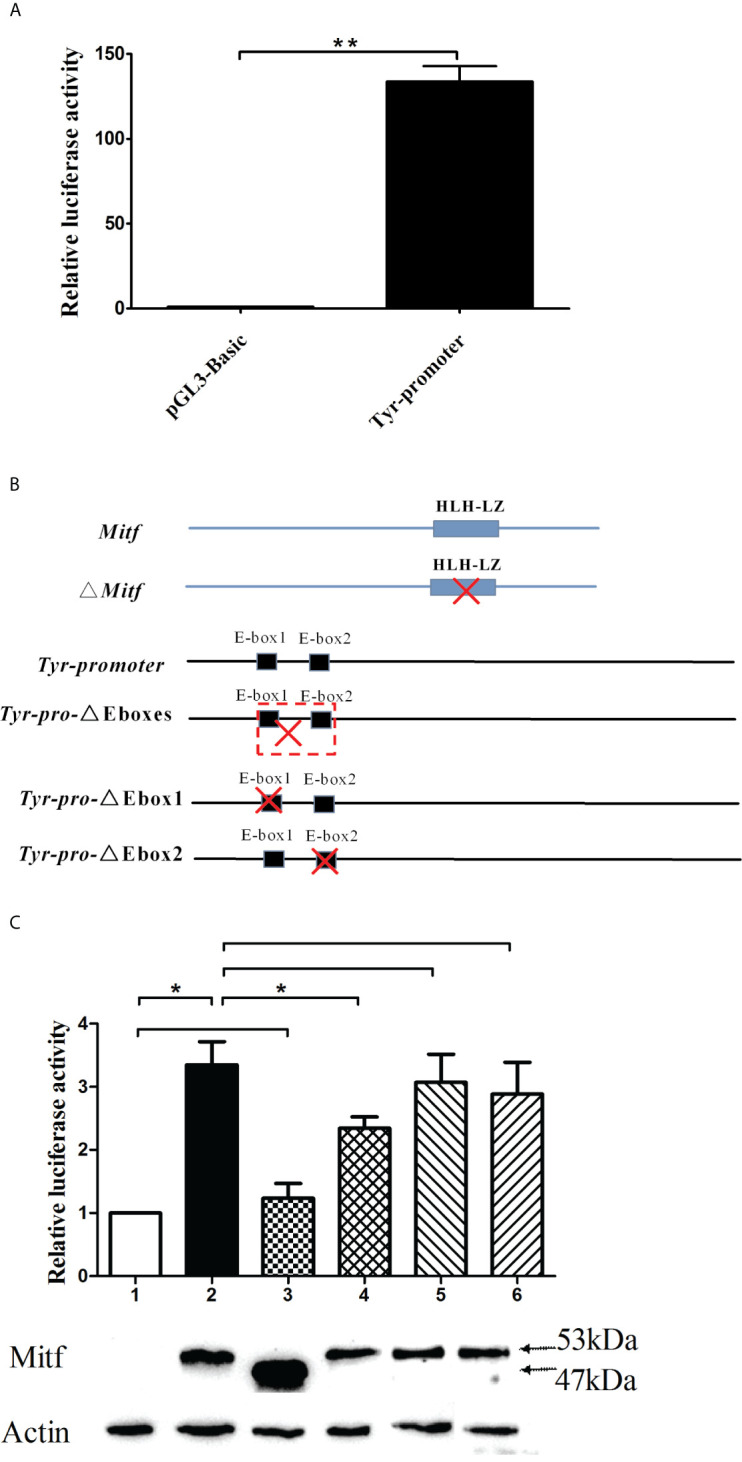
The *PpTyr* promoter activity was induced by *PpMitf*. **(A)** *PpTyr* promoter activity analysis. The 293T cells in 24-well plates were transfected with 0.4 μg of Tyr-promoter-Luc (pGL3-Basic in control) and 0.04 μg pRL-cmv vector. 48 h post transfection, cells were collected for luciferase activity assays. **(B)** The construction of *Mitf*-pcDNA3.1, Mitf-△HLHLZ-pcDNA3.1, Tyr-△Eboxes-promoter-Luc, Tyr-△Ebox1-promoter-Luc and Tyr-△Ebox2-promoter-Luc plasmids. **(C)** The function analysis of HLHLZ domain in MITF and E-box in Tyr promoter by luciferase activity analysis and western blot. Column and lane 1, the 293T cells were cotransfected with pcDNA3.1 and Tyr-promoter-Luc vector; Column and lane 2, *Mitf*-pcDNA3.1 and Tyr-promoter-Luc; Column and lane 3, Mitf-△HLHLZ-pcDNA3.1 and Tyr-promoter-Luc; Column and lane 4, *Mitf*-pcDNA3.1 and △Eboxes-promoter-Luc; Column and lane 5, *Mitf*-pcDNA3.1 and △Ebox1-promoter-Luc; Column and lane 6, *Mitf*-pcDNA3.1 and △Ebox2-promoter-Luc. The data were represented as the mean ± SD (*N* = 5). *P <0.05; **P <0.01; No signal means no difference.

The activity of *PpTyr* promoter was measured by dual-luciferase reporter assays. The Tyr-promoter-Luc (from −1,943 to -1) was constructed and transfected into the 293T cells. The pGL3-Basic vector was transfected as control. As shown in **Figure 8A**, cells transfected with pGL3-Basic vector showed a low level of luciferase activity, while the Tyr-promoter-Luc construct induced a high luciferase activity, indicating that this is a strong promoter.

To investigate whether *PpMitf* regulated the expression of *PpTyr*, the 293T cells were cotransfected with Tyr-promoter-Luc plasmid and *Mitf*-pcDNA3.1 plasmid, or empty plasmid pcDNA 3.1 as control. The luciferase activities analysis showed that overexpression of *Mitf* yielded an increasing luciferase activity, being 3.02 fold of pcDNA 3.1 control cells (*P <*0.05). The *Mitf*-△HLHLZ-pcDNA3.1 plasmid was constructed and used to analyze the function of HLH-LZ domain. The overexpression of *Mitf* without HLH-LZ domain only yielded 23.3% increase (*P >*0.05) in luciferase activities compared to pcDNA 3.1 control, but yielded 63.2% decrease (*P <*0.05) compared to *Mitf*-pcDNA3.1 group (**Figures 8B, C**). The data indicated that *Mitf* was able to activate the expression of *Tyr*, and the HLH-LZ was the key functional domain of MITF.

To elaborate the important role of E-box in *Tyr* promoter, the Tyr-△Ebox1-promoter-Luc, Tyr-△Ebox2-promoter-Luc and Tyr-△Eboxes-promoter-Luc plasmids were constructed for luciferase activity assay. The deletion from E-box1 to E-box2 yield 30.2% decrease in luciferase activity compared to *Tyr* promoter group (*P <*0.05), but single E-box1 or E-box2 deletion failed to change luciferase activity (**Figures 8B, C**). These data indicated that the regions from −1,767 to −1,607, where two E-box domains were located, were important for *Tyr* promoter activity and *Mitf* regulation.

To confirm the successful overexpression of MITF protein in 293 cells, these transfected cells were collected for western blot detection. Because there was no endogenous expression of *Mitf* in 293 cells, The MITF protein level in pcDNA3.1 group was close to 0. All cells transfected with *Mitf*-pcDNA3.1 plasmid had high levels of MITF protein, whose molecular weight was about 53 kDa, including the Flag-tag. Cells transfected with Mitf-△HLHLZ-pcDNA3.1 plasmid also had a high expression level of MITF-△HLHLZ protein, whose molecular weight was about 47 kDa (**Figure 8C**).

### *PpMitf* Silencing Inhibited Antibacterial Activity of Hemolymph Supernatant in *P. penguin*

**Figure 9:**
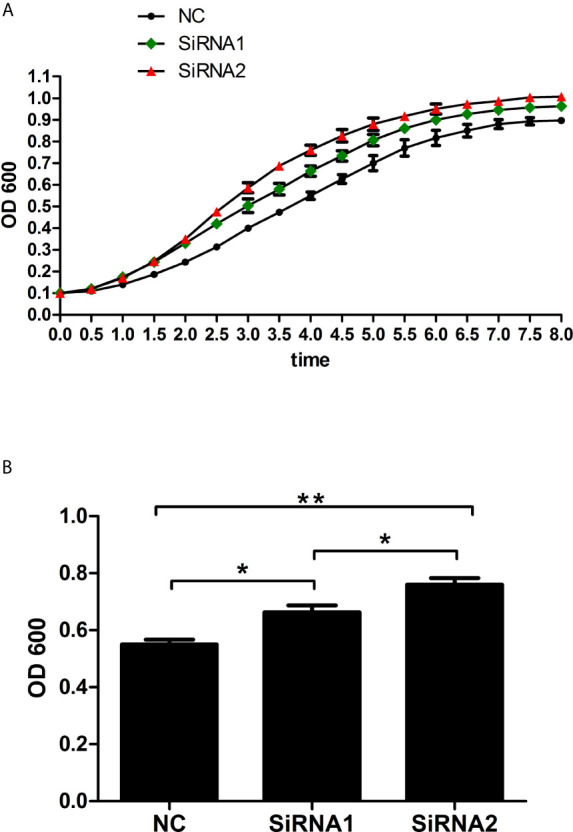
Antibacterial activity of haemolymph supernatant from samples after *PpMitf* RNAi. **(A)** Growth curves of *E. coli* exposed to haemolymph supernatants of NC, siRNA1 and siRNA2 groups. **(B)** OD600 value of *E. coli* at *T50* in different groups. Each value was shown as mean ± SD (*N* = 5). *P <0.05; **P <0.01.

Since *Mitf* activated the expression of *Tyr*, which was known to play crucial roles in innate immunity of vertebrate (27), we speculated that *PpMitf* participated in innate immunity of *P. penguin*. After *PpMitf* silencing, the antimicrobial activity of hemolymph supernatant was measured and represented by its inhibition effect on *E. coli* growth. Because the OD600 absorbance of NC group reached the maximum at 8h, 4h was defined as T50 (**Figure 9A**). As shown in **Figure 9B**, in siRNA1 group, the values of OD600 was 0.71 at 4 h, significantly up-regulated by 20.6% compared to that of NC group (*P <*0.05), which was 0.55. The OD600 value was 0.75 in siRNA2 group, significantly increased by 38.2% than NC group (*P <*0.05). This indicated that the antibacterial activity of hemolymph supernatants were inhibited by *Mitf* silencing.

### The Antibacterial Activity Was Inhibited by Decreasing Melanin Content Resulted from *PpMitf* Silencing

**Figure 10:**
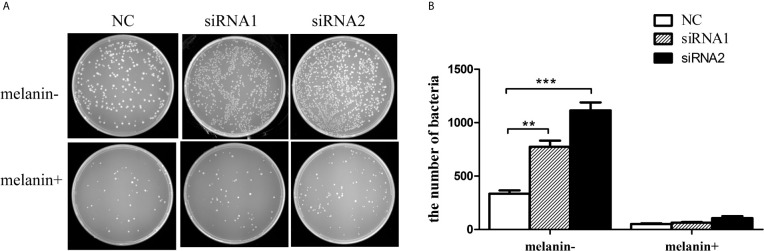
Antibacterial activity of melanin oxidation products from *P. penguin* samples. **(A)** Photographs showed the number of *E. coli* after *Mitf* silencing and adding melanin oxidation products. **(B)** Histogram showed the number of *E. coli* after *Mitf* silencing and adding melanin oxidation products. Each value was shown as mean ± SD (*N* = 5; *P <0.05; **P <0.01).

To detect whether the decrease of antibacterial activity was directly related with the melanin, we investigated the anti-bacteria effect of melanin oxidation products from *P. penguin* samples. **Figures 10A, B** showed that the numbers of bacteria were sharply increased by 135.3% (*P <*0.01) and 240.7% (*P <*0.001) in siRNA1 and siRNA2 groups compared to the NC group. In contrast, by adding exogenetic melanin oxidation production, the number of bacteria was decreased by 84.5% (*P <*0.01) in NC group, 91.9% (*P <*0.001) in siRNA1 group and 90.5% (*P <*0.001) in siRNA2 group. The data demonstrated melanin oxidation production from mantle of *P. penguin* had the antibacterial activity, and the decrease of melanin content resulted from *PpMitf* silencing was a direct reason for decline of antibacterial activity.

## Dicussion

The global pearl culture industry faces two main problems, how to make the pearl colorful and how to resist the serious disease caused by pathogenic infections. The study on mechanism of color formation and immune response will be very helpful for solving the two main problems. Melanin widely exists in vertebrate and plays many role including pigmentation, anti-ultraviolet radiation and would healing (28). Therefore, we speculated melanin, which had been verified to have decisive role in color formation (15, 17), also participated in innate immunity in bivalves. The melanin synthesis pathway and innate immunity pathway might be interactive and interdependent, and some genes might involve in innate immune response by regulating the production of melanin.

MITF is responsible for the normal development of several cell lineages (29, 30). In vertebrates, MITF is a key regulator in melanin synthesis pathway, and controls the differentiation, proliferation, migration and survival of melanocytes (31–33). Meanwhile, MITF involves in immune defense by regulating a series of immune-related genes (7, 11, 12). In this study, a novel *Mitf* gene from *P. penguin* was identified, and its functions were deliberated by RNA interference. The *PpMitf* knockdown significantly reduced tyrosinase activity and melanin content, indicating that *PpMitf* participated the melanin synthesis of *P. penguin*. Meanwhile, the *PpMitf* knockdown also apparently decreased the antibacterial activity *of* hemolymph supernatant, indicating that it played a crucial role in innate immune defense of *P. penguin.* The *Mitf* was considered to be a bifunctional regulator in both melanin synthesis pathway and innate immunity pathway of *P. penguin*.

MITF contains a basic helix-loop-helix-leucine zipper (bHLH-LZ) domain that binds DNA as dimers (34). In this research, multiple sequence alignments showed the *Mitf* of *P. penguin* was conserved with *Mitf* genes from other species, and the highest homology was found in bHLH-LZ domain as expected. Moreover, a relatively conserved region presented in the N-terminal of *PpMitf* and was named as “N-terminal conserved domain”. Because N-terminal conserved domain widely existed in all MiT-TFE family members, including transcription factor EB (TFEB), transcription factor EC (TFEC), transcription factor E3(TFE3) and MITF (35), we speculated it was important for transcription factor to play the role of transcription regulation. So we respectively silenced the bHLH-LZ and N-terminal conserved domain by RNA interference to investigate their roles. Silencing of each domain apparently inhibited tyrosinase activity, melanin content, related-genes’ expression and antibacterial activity, indicating both bHLH-LZ domain and N-terminal conserved domain were important for the function of *Mitf* gene. Furthermore, the bHLH-LZ domain silencing had more significant inhibition effect on melanin synthesis and innate immunity of *P. penguin*, which indicated bHLH-LZ domain was the key domain for *Mitf* gene.

Tyrosinase is a monophenol monooxygenase, which can catalyze the hydroxylation of phenols to catechols and the oxidation of catechols to quinones (23, 36, 37), and is considered as the initial and rate-limiting enzyme for melanin production in both vertebrate (9, 10) and invertebrate (38, 39). Meanwhile, tyrosinase also is known for its role in would healing, radiation protection, primary immune responses due to its phenoloxidase activity (40, 41). In vertebrate, by eletromobility shift assays (EMSA), chromatin immunoprecipitation (ChIP) and reporter assays, many studies showed MITF directly regulated the expression of tyrosinase gene (42). In this study, the promoter sequence of tyrosinase was amplified and a *Tyr* promoter-driven luciferase reporter construct was made for luciferase activity analysis. The overexpression of *Mitf* significantly increased luciferase activities of *Tyr*-promoter, indicating that *Mitf* functioned by activating the expression of *Tyr* in *P. penguin.* However, the bHLH-LZ deleted MITF failed to activate the *Tyr* promoter, indicating that the bHLH-LZ domain was a critical functional domain of MITF protein. The results was consistent with bHLH-LZ RNA interference data, which showed a significant inhibition effect on melanin synthesis and immunity capability by bHLH-LZ domain silencing in *P. penguin*.

Moreover, two typical E-box (CATGTG) were found to locate at positions from −1,767 to −1,761 and from −1,613 to −1,607 in *PpTyr*-promoter. Previous studies reported that the basic region of bHLH-LZ of MITF bound to E-box (CAC/TGTG) or M-box (a core CATGTG with additional flanking residues) in the promoter of targeted genes as a homodimer or heterodimer in vertebrate (34). So the functions of E-box were analyzed in *P. penguin*. The data showed that single knockdown of E-box1 or E-box2 could not significantly inhibit the luciferase activity of *Tyr* promoter. This result was inconsistent with previous study, which reported that each of 3 E-box in tyrosinase promoter could specially bind to the MITF in mouse (43). A possible reason was that the E-box was too far from transcription start site (TSS), and weakened its role in *Tyr* promoter of *P. penguin*. Fortunately, both E-box deletions significantly inhibited *Tyr* promoter activity, indicating the critical role of region including E-box1 and E-box2. A synergistic effect was speculated to exist between E-box1 and E-box2, which enhanced the role of single E-box and strengthened the activating of *Mitf* to *Tyr* promoter (44).

Since *Mitf* was considered to involve in immune response by tyrosinase-mediated melanin pathway in *P. penguin*, we wondered whether melanin itself took part in immune response. In this study, the antibacterial activity of melanin oxidation products from different groups were detected. By *PpMitf* silencing, the melanin content was significantly decreased, and the number of bacteria was significantly increased. Oppositely, by adding melanin oxidation products, the inhibition effect on bacteria growth of different groups was apparently recovered. Our data was supported by these reports, which demonstrated that PDCA was a good antibacterial compound (45), and PTCA had anti-inflammatory and anti-oxidation properties (46). The results indicated that melanin itself directly involved in innate immunity, and the *Mitf-tyrosinase-*melanin pathway played an important role in innate immune system of *P. penguin.*

After *Mitf* silencing, the expression of three downstream genes were analyzed, including *Tyr*, *Cdk2* and *Bcl2*. *Tyr* is a key rate-limiting enzyme of melanogenesis by catalyzing three important reactions, and controls the speed of melanin synthesis (16). *Cdk2* is known for its function in cell cycle, and plays an important role in controlling melanoma growth (24). *Bcl2* is an anti-apoptotic gene, and takes part in controlling melanoma survival (47). *Mitf* was reported to regulate the transcriptional activity of *Tyr*, *Cdk2* and *Bcl2* by binding to their promoters in vertebrate (16, 24, 45). Our data showed that the *Mitf* silencing significantly inhibited the *Tyr*, *Cdk2* and *Bcl2* transcripts, and suggested that *Mitf* might involve in melanin synthesis, melanocyte growth and melanocyte survival in *P. penguin.* Moreover, *Cdk2* and *Bcl2* themselves also play an important part in immune response. *Cdk2* controlled peripheral immune tolerance, promoted T cell differentiation and restricted Treg function in immune responses (48). *Bcl2* was named B cell lymphoma/leukemia-2 gene, whose mutation leaded to serious apoptosis (49, 50). The reduction of *Cdk2* and *Bcl2* transcripts after *Mitf* silencing in *P. penguin* suggested that *Mitf* might participate in innate immunity by regulating the expression of *Bcl2* and *Cdk2* genes in another immune response pathway, in addition to the melanin synthesis pathway.

In conclusion, a novel *Mitf* gene was characterized from *P. penguin.* The polypeptide sequence alignment showed a highly conserved bHLH-LZ domain. Tissue distribution analysis revealed that *PpMitf* was highly expressed in mantle and heamocytes, tissues responsible for color formation and innate immunity. *PpMitf* silencing significantly decreased the tyrosinase activity, melanin content, immune-related genes’ expression and antibacterial activity, indicating that *PpMitf* involved in both melanin synthesis and innate immunity of *P. penguin.* The promoter analysis and luciferase activity analysis showed that MITF regulated melanin synthesis by activating the E-box in *Tyr* promoter through highly conserved bHLH-LZ domain in MITF. The antibacterial activity analysis revealed that melanin, which was regulated by *Mitf*, had direct antibacterial effect. The study demonstrated that *PpMitf* played a key role in innate immunity through activating tyrosinase-mediated melanin synthesis in *P. penguin.* Our findings have offered important insights for molecular mechanism of innate immunity in pearl shell.

## Data Availability Statement

The datasets presented in this study can be found in online repositories. The names of the repository/repositories and accession number(s) can be found in the article/supplementary material.

## Author Contributions

XY and FY designed the experiments. FY performed experiments and wrote the manuscript. YL and BQ analyzed data. ZZ and JC contributed to the graphing. MW offered the experimental animals. XY and YL revised the manuscript. All authors contributed to the article and approved the submitted version.

## Funding

This work was supported by National Key R&D Program of China (2019YFD0900800), Natural Science Foundation of Guangdong Province (2021A1515011052), Guangdong Innovative and Strong School Project (230419094), and Guangdong Marine Fishery Development Foundation (B201601-Z08).

## Conflict of Interest

The authors declare that the research was conducted in the absence of any commercial or financial relationships that could be construed as a potential conflict of interest.
